# Case Report: Ruxolitinib for systemic juvenile idiopathic arthritis complicated by macrophage activation syndrome: two pediatric cases and literature review

**DOI:** 10.3389/fped.2026.1812770

**Published:** 2026-05-28

**Authors:** Honglin Liu, Zhigang Wang, Wei Zhang

**Affiliations:** 1 Pediatric Immunology and Rheumatology Department, Chengdu Women's and Children's Central Hospital, School of Medicine, University of Electronic Science and Technology of China, Chengdu, China; 2 Radiology Department, Chengdu Women's and Children's Central Hospital, School of Medicine, University of Electronic Science and Technology of China, Chengdu, China

**Keywords:** JAK inhibitor, macrophage activation syndrome, plasma exchange, ruxolitinib, salvage therapy, systemic juvenile idiopathic arthritis

## Abstract

**Objective:**

To explore the efficacy and safety of ruxolitinib in children with systemic juvenile idiopathic arthritis complicated by macrophage activation syndrome (sJIA-MAS), particularly in those with refractory disease.

**Methods:**

The clinical courses of two children with refractory sJIA-MAS treated with ruxolitinib at our center were retrospectively reviewed. In addition, Chinese and English databases were searched to summarize the clinical features, therapeutic outcomes, and safety of ruxolitinib in pediatric sJIA-MAS.

**Results:**

In both patients from our center, systemic hyperinflammation improved markedly during combined therapy that included ruxolitinib, allowing glucocorticoids to be gradually tapered and discontinued without relapse. Case 1 was a 19-month-old boy diagnosed with refractory sJIA-MAS complicated by respiratory failure. Despite methylprednisolone pulse therapy, low-grade fever and rash persisted. After starting ruxolitinib, body temperature stabilized and clinical symptoms improved rapidly. Glucocorticoids were discontinued within three months, and tocilizumab was stopped after one year. Case 2 was a 4-year-old girl diagnosed with refractory sJIA-MAS complicated by acute respiratory distress syndrome and thrombocytopenia. She received methylprednisolone pulse therapy and ruxolitinib combined with plasma exchange (three sessions). Body temperature normalized on the second day after ruxolitinib initiation. Dyspnea resolved, and ferritin levels normalized within 10 days. Glucocorticoids were discontinued within six months. No ruxolitinib-related adverse events were observed. In addition, five previously reported pediatric cases were identified. A total of seven children (three males and four females; age range 1–11 years) were analyzed. Of the seven patients reviewed, five met criteria for refractory sJIA-MAS, while two were not strictly refractory but received ruxolitinib due to incomplete response or intolerance to standard therapy. Five achieved complete remission and two achieved partial remission. Two patients experienced relapse during glucocorticoid tapering and achieved complete remission after the addition of canakinumab. No deaths were reported. One patient developed Epstein–Barr virus–associated hemophagocytic lymphohistiocytosis and neutropenia after ruxolitinib treatment, which resolved after drug discontinuation and plasma exchange.

**Conclusion:**

Ruxolitinib may serve as a potential rescue or bridging therapy for patients with sJIA-MAS, especially those with refractory disease unresponsive to first-line therapy. Close monitoring for cytopenia and viral infections is recommended during treatment.

## Introduction

Systemic juvenile idiopathic arthritis (sJIA) is a subtype of juvenile idiopathic arthritis characterized by persistent fever, evanescent rash, and marked systemic inflammation, accounting for approximately 10% of cases (1). Macrophage activation syndrome (MAS) is one of its most serious complications, occurring in an estimated 7%–14% of patients (2–4). Subclinical or occult MAS may be present in up to 30%–40% of cases (5), and reported mortality rates range from 8% to 22% (6). High-dose glucocorticoids are the mainstay of initial treatment for sJIA-MAS and are commonly used in combination with cyclosporine A or biologic agents targeting interleukin (IL)-1 or IL-6. However, some patients show an incomplete or unsustained response, with ongoing or recurrent hyperinflammation (7–9). In this setting, additional therapies such as etoposide, Janus kinase (JAK) inhibitors, or plasma exchange have been used to achieve better disease control and reduce the risk of organ dysfunction (9). The 2024 EULAR/PReS recommendations for the management of Still's disease also advocate individualized treatment for MAS that does not respond to standard therapy, while noting the limited availability of high-quality evidence (7).

Ruxolitinib, a selective JAK1/2 inhibitor, has been described in recent case reports and small series of patients with sJIA-MAS and has emerged as a potential option when first-line therapy is insufficient (10, 11). Nevertheless, experience in pediatric populations remains limited. Here, we report two pediatric cases of refractory sJIA-MAS successfully treated with ruxolitinib and summarize the available pediatric literature to further evaluate its efficacy and safety.

This study was approved by the Ethics Committee of Chengdu Women's and Children's Central Hospital [approval number: KY-2025(21)] and was granted a waiver of written informed consent from the patients' guardians.

### Case 1

A 1-year-7-month-old boy was admitted on September 15, 2022, with an 11-day history of fever. His maximum temperature reached 40 °C, presenting as a remittent pattern. Scattered erythematous maculopapular rashes appeared across his body during febrile episodes and faded with defervescence. At onset, physical examination showed no hepatosplenomegaly, lymphadenopathy, or joint abnormalities. Neither patient had recurrent or severe infections, and there was no family history of rheumatic, immunodeficiency, or hemophagocytic lymphohistiocytosis(HLH) disorders. Laboratory tests revealed a white blood cell count (WBC) of 15.12 × 10⁹/L, hemoglobin (Hb) 102 g/L, platelet count (PLT) 483 × 10⁹/L, high-sensitivity C-reactive protein (hs-CRP) 25.29 mg/L, erythrocyte sedimentation rate (ESR) 88 mm/h, D-dimer 1.35 μg/mL, and fibrinogen(FIB) 4.34 g/L, triglyceride(TG) 1.16 mmol/L, and the Aspartate aminotransferase (AST) was 82 U/L and lactate dehydrogenase (LDH) was 672 U/L. Autoimmune serological testing, including antinuclear antibody (ANA) and sixteen specific autoantibodies (such as anti-dsDNA, anti-Sm, anti-SSA/Ro52, anti-SSA/Ro60, anti-SSB/La, anti-Scl-70, and so on), was performed, and all results were negative. Bone marrow cytology was normal. Extensive infectious work-up (cultures, viral PCR, tuberculosis screening, and mNGS) showed no active infection. Chest CT revealed pulmonary inflammation with mediastinal and hilar lymphadenopathy, and abdominal CT demonstrated mesenteric lymph node enlargement without hepatosplenomegaly. Echocardiography revealed left coronary artery dilation (LCA 2.6 mm, Z = 2.36; LAD 2.0 mm, Z = 1.51) (12). Incomplete Kawasaki disease (KD) refractory to intravenous immunoglobulin (IVIG) was initially suspected. The patient received two courses of IVIG (2 g/kg each), but the fever persisted. Trio whole-exome sequencing revealed no pathogenic variants in genes associated with primary hemophagocytic lymphohistiocytosis, and primary HLH was further excluded based on normal immunological screening results. On September 19, 2022, after more than two weeks of persistent fever, evanescent erythematous rash, generalized lymphadenopathy, peripheral leukocytosis (> 15.12 × 10⁹/L), and exclusion of infection, malignancy, autoimmune disease, and monogenic autoinflammatory disorders, he was diagnosed with sJIA according to the 2019 PRINTO classification criteria (13). Oral prednisone acetate was started at 2 mg/kg/day in divided doses; however, fever did not improve significantly.

**Figure 1:**
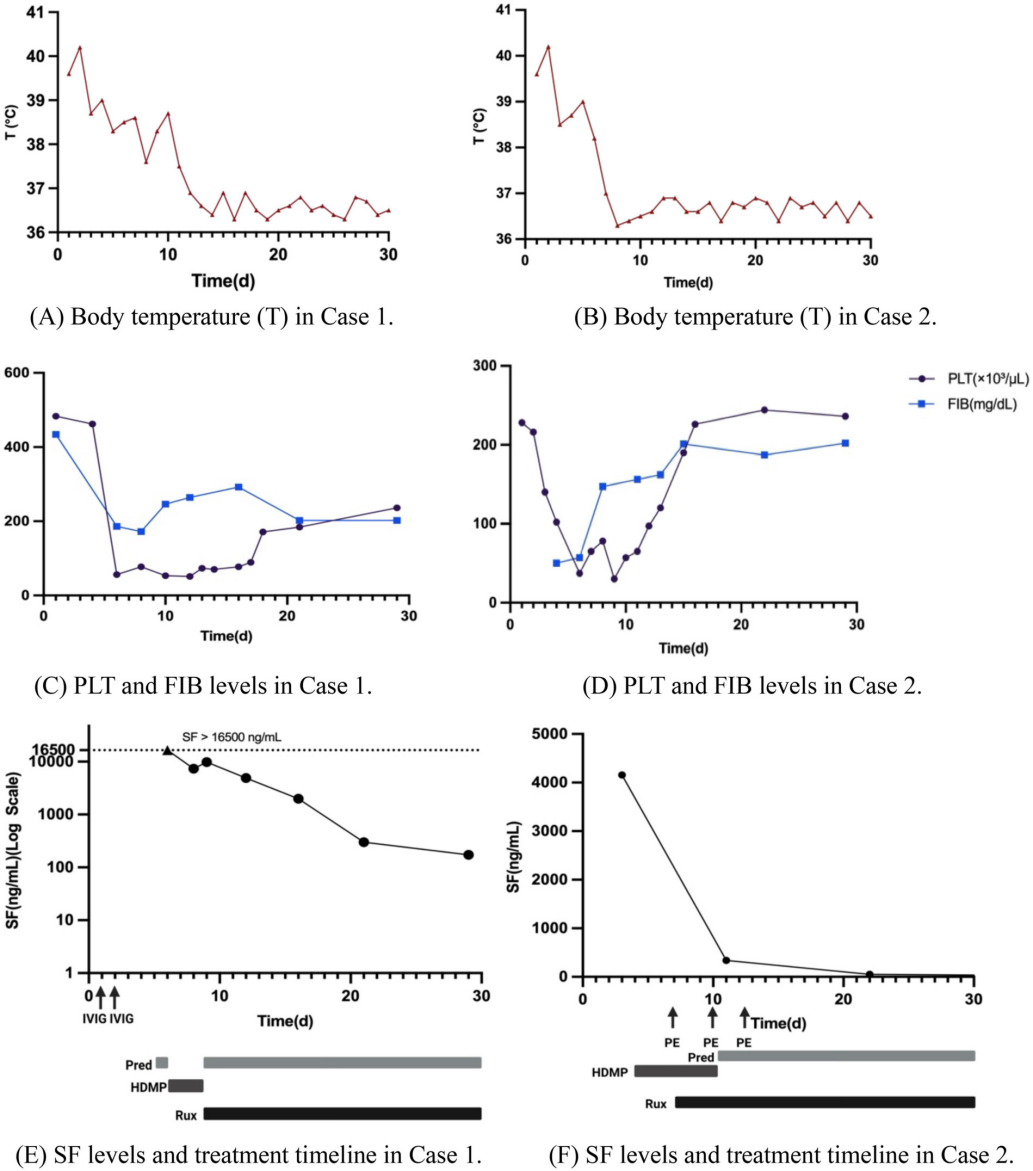
Treatment course and changes in clinical indicators in two cases during hospitalization for MAS. **(A,B)** Body temperature (T) curves for Case 1 and Case 2, respectively. **(C,D)** Dynamic changes in PLT and FIB levels. **(E,F)** SF levels and treatment timelines. PE: three consecutive sessions were performed, each processing approximately 1.0–1.5 plasma volumes (∼1,000 mL), using fresh frozen plasma (FFP) as replacement fluid. PLT, platelet; FIB, fibrinogen; SF, serum ferritin; IVIG, intravenous immunoglobulin; HDMP, high-dose methylprednisolone; Pred, prednisone; PE, plasma exchange; Rux, ruxolitinib.

On September 20, 2022, repeat tests showed WBC 2.6 × 10⁹/L, Hb 78 g/L, PLT 57 × 10^9/L, CRP 20.9 mg/L, ferritin(SF) > 16,500 ng/mL, AST 1,167 U/L, FIB 1.86 g/L, and TG 2.87 mmol/L. Repeat chest and abdominal CT revealed lymphadenopathy and hepatosplenomegaly. Repeat bone marrow examination on that day revealed no hemophagocytosis. Based on the 2016 EULAR/ACR/PRINTO criteria (14), MAS was diagnosed. Methylprednisolone pulse therapy (15 mg/kg/day) was given for 3 days. Although blood counts improved, the patient continued to have low-grade fever, recurrent rash, persistently elevated ferritin, and worsening liver function, indicating refractory sJIA-MAS. After discussion with the family and obtaining written informed consent, ruxolitinib (5 mg twice daily) was started. Intravenous methylprednisolone was stopped and replaced with oral prednisone acetate (2 mg/kg/day). Three days after starting ruxolitinib, the patient's body temperature normalized, and the rash resolved. Ferritin, liver function, coagulation parameters, and lipid levels gradually returned to normal. The overall laboratory trends are shown in Figure 1. Follow-up echocardiography in December 2022 demonstrated complete normalization of coronary dimensions. Tocilizumab was added on October 22, 2022, and prednisone was tapered and discontinued within 3 months. Tocilizumab was stopped one year later. Ruxolitinib was gradually tapered and discontinued in January 2024. During follow-up to date, no relapse has occurred.

### Case 2

A 4-year-old girl was admitted on May 25, 2024, with a 2-month history of persistent rash and 5 days of fever. Her maximum temperature reached 40.5 °C, presenting as a remittent pattern. Scattered erythematous rashes of varying sizes appeared across her body during febrile episodes and faded after defervescence. She reported pain in both hips, knees, and ankles, which worsened during fever and improved afterward. Physical examination revealed splenomegaly and lymphadenopathy. No tenderness or swelling was noted in peripheral joints. There was no history of recurrent severe infections, and no family history of rheumatic disease, immunodeficiency, or hemophagocytic lymphohistiocytosis. Laboratory tests showed WBC 15.91 × 10⁹/L, hs-CRP 147.3 mg/L, PLT 221 × 10⁹/L, ESR 49 mm/h, D-dimer 7.12 μg/mL, FIB 6.61 g/L, SF 1,400.1 ng/mL, and normal liver enzymes. Autoimmune screening was negative, using the same comprehensive 16-antibody ANA-associated panel as described in Case 1, and rheumatoid factor was also negative. Joint ultrasonography was unremarkable. The patient was initially treated with cefoperazone-sulbactam for presumed infection, but fever persisted. Incomplete KD refractory to IVIG was considered. She received two courses of IVIG (2 g/kg each) and methylprednisolone sodium succinate (2 mg/kg/day), followed by oral prednisone acetate (1 mg/kg/day). Despite these therapies, remittent fever persisted for 14 consecutive days, temporarily stabilizing for 4 days before rising again. WBC increased to 52.11 × 10^9/L. On June 9, 2024, after persistent fever for more than 2 weeks, arthritis, evanescent erythematous rash, generalized lymphadenopathy, hepatosplenomegaly, and peripheral leukocytosis (> 15.12 × 10⁹/L), with exclusion of infection, malignancy, autoimmune disease, and monogenic autoinflammatory disorders, she was diagnosed with sJIA according to the 2019 PRINTO classification criteria (13). Tocilizumab was initiated the same day, and body temperature normalized within four days. After discharge, she continued oral prednisone (1 mg/kg/day), regular tocilizumab infusions, and methotrexate therapy.

**Figure 2:**
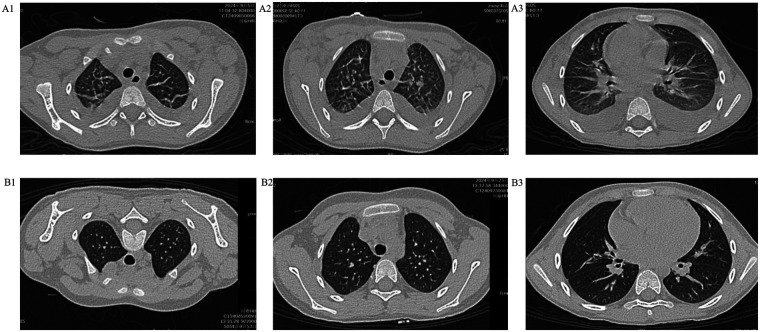
Chest computed tomography images of case 2. **(A)** Bilateral interstitial lung involvement with prominent pulmonary edema during active macrophage activation syndrome. **(B)** Marked absorption of interstitial lesions and pulmonary edema two weeks after initiation of ruxolitinib and plasma exchange.

On September 2, 2024, she developed sudden high fever, new rash, lethargy, and tachypnea (60–70 breaths/min). Laboratory tests revealed WBC 51.97 × 10^9/L, absolute neutrophil count (ANC) 48.51 × 10⁹/L, PLT 140 × 10⁹/L, hs-CRP 4 mg/L, FIB 0.5 g/L, SF 4,157 ng/mL, AST 846.7 U/L, TG 3.2 mmol/L, interleukin-6 (IL-6) 3,346.65 pg/mL, interleukin-10(IL-10) 244.86 pg/mL, and interferon-*γ* (IFN-*γ*) 319.23 pg/mL. Chest CT showed bilateral interstitial pulmonary edema with small-to-moderate pleural effusion. At the onset of MAS, repeat infectious evaluation was performed, including blood cultures and viral screening. EBV and human herpesvirus-6 (HHV-6) testing was negative, and no evidence of active infection was identified. Trio whole-exome sequencing revealed no pathogenic variants in genes associated with primary hemophagocytic lymphohistiocytosis. MAS was diagnosed on September 5, 2024, according to the 2016 EULAR/ACR/PRINTO criteria (14).She received methylprednisolone sodium succinate pulse therapy (21.7 mg/kg/day) for 3 days. During this period, further evaluation was undertaken to exclude primary hemophagocytic lymphohistiocytosis (HLH). However, tachypnea persisted, transcutaneous oxygen partial pressure remained 70–75 mmHg, and PLT decreased to 37 × 10⁹/L, suggesting refractory sJIA-MAS. After discussion with the family and obtaining written informed consent, ruxolitinib (10 mg twice daily) was initiated on September 8, 2024, along with three sessions of plasma exchange. Four days later, tachypnea improved, and PLT and FIB gradually returned to normal. SF also decreased significantly. The overall laboratory trends are shown in Figure 1. Her body temperature stabilized, and the joint pain and rash resolved. Two weeks later, repeat laboratory tests showed WBC 8.49 × 10⁹/L, PLT 190 × 10⁹/L, hs-CRP < 0.5 mg/L, fibrinogen 2.01 g/L, ferritin 26.7 ng/mL, AST 24.5 U/L, TG 1.47 mmol/L, and ESR 7.3 mm/h, indicating complete remission of sJIA-MAS. A follow-up low-dose chest CT performed at the same time demonstrated marked resolution of interstitial pulmonary edema (Figure 2). Glucocorticoids were subsequently tapered and discontinued within 6 months. During treatment, she developed transient neutropenia (1.12 × 10^9/L), but Epstein–Barr virus monitoring remained negative. She continues to receive regular tocilizumab infusions, and ruxolitinib has been tapered to 2.5 mg twice daily. No relapse has occurred during follow-up.

## Methods

### Literature search and study inclusion

A literature search was performed in PubMed using the terms “systemic juvenile idiopathic arthritis,” “macrophage activation syndrome,” and “ruxolitinib.” For Chinese-language databases (CNKI, Wanfang, VIP), the corresponding Chinese search terms were applied, which were the Chinese translations of the English keywords used in PubMed. The search covered publications from database inception to June 2025. After removing duplicates and records without full text, three studies with complete clinical data were included. Together with two cases from our center, a total of seven pediatric patients with refractory sJIA-MAS were analyzed.

### Definition of refractory sJIA-MAS

Refractory sJIA-MAS describes MAS in sJIA patients who do not achieve lasting clinical or laboratory improvement following adequate treatment with high-dose glucocorticoids and other standard immunosuppressive or biologic therapies, with ongoing, progressive, or recurrent disease activity (8, 15).

### Assessment of MAS treatment response

As no universally accepted response criteria for MAS have been established, treatment response was assessed based on predefined clinical and laboratory parameters. Treatment response was classified as complete remission (CR) or partial remission (PR). CR was defined as resolution of fever and other MAS-related clinical manifestations, together with normalization of key laboratory abnormalities, including ferritin, platelet count, aspartate aminotransferase (AST), triglycerides, and fibrinogen, according to institutional reference ranges. PR was defined as clinical improvement accompanied by a ≥ 25% reduction in at least two abnormal laboratory parameters compared with baseline, without full normalization (16, 17).

### Assessment of sJIA disease activity

Disease activity of sJIA was assessed according to the 2011 American College of Rheumatology (ACR) provisional criteria for clinical inactive disease (Wallace criteria). Clinical inactive disease was defined as the absence of active arthritis; no fever, rash, serositis, splenomegaly, or generalized lymphadenopathy attributable to sJIA; normal ESR and/or CRP; and a physician's global assessment indicating no disease activity (18).

### Assessment of pulmonary involvement

Pulmonary involvement was assessed using clinical manifestations and chest computed tomography (CT) findings. Improvement was defined as relief of respiratory symptoms accompanied by a marked reduction or resolution of pulmonary abnormalities on follow-up CT compared with baseline. All imaging studies were reviewed by an experienced radiologist, and assessments were performed at follow-up after clinical stabilization following treatment.

## Results

Through a retrospective review, we summarized the clinical characteristics, laboratory findings, efficacy, and safety of ruxolitinib in seven pediatric patients with sJIA-MAS, including the two cases from our center (Table 1) (15, 19, 20). Of these seven cases, five were refractory sJIA-MAS, while two were not strictly refractory: one patient had inadequate response to tocilizumab and experienced glucocorticoid-related adverse effects, and the other lacked information regarding prior high-dose glucocorticoid therapy failure. Among all seven children (three males and four females; age range 1–11 years), fever was present in all cases (100%), rash occurred in 4/7 patients, hepatosplenomegaly in 4/7, and arthritis in 4/7, mainly involving the bilateral proximal femur, ankles, and knees. Edema was reported in 2/7 patients (one with generalized edema and one with dorsal foot edema). Respiratory failure occurred in 2/7 patients, and coronary artery dilation was observed in 1/7.Laboratory abnormalities included hyperferritinemia (100%), elevated liver enzymes (100%), hypofibrinogenemia (100%), and thrombocytopenia (4/7).

**Table 1:** Clinical characteristics, treatment details, and outcomes of seven pediatric patients with sJIA-MAS treated with ruxolitinib.

Author (year)	Patients	Sex	Age (years)	Timing of MAS	Main clinical characteristics	Biochemical alterations	Treatment before ruxolitinib	Concomitant treatments at ruxolitinib initiation	Treatments at the last follow-up	Ruxolitinib dosing	Ruxolitinib duration (months)	Follow-up after ruxolitinib	Other adverse side effects at ruxolitinib duration
Irabu et al. (2025) (20)	P1	Female	3	At sJIA onset	Fever Rash Arthritis	FER↑ AST↑ Coagulopathy	Methylprednisolone pulse (30 mg/kg/day) Dexamethasone palmitate CsA Tocilizumab Canakinumab	Prednisone (40 mg/day) CsA	Not described	10 mg/day→7.5 mg/day→10 mg/day→7.5 mg/day	NR	Complete remission	7 days ： neutropenia↓, lymphopenia ↓ 3 weeks: EBV-HLH
Wu et al. (2025) (19)	P2	Female	11	Active sJIA (recurrent MAS)	Fever	WBC↓ PLT↓ ALT ↑ AST↑ FBI ↓ FER ↑	Methylprednisolone pulse(25 mg/kg/day) Tocilizumab CsA	Methylprednisolone pulse (20 mg/kg/day×3 days) followed by prednisone (40 mg/day) CsA (2.8 mg/kg/d)	Not described	5 mg bid→10 mg bid	3	Complete remission：3 months Improvement Steroid reduction	Not described
He et al. (2023) (15)	P3	Male	7	At sJIA onset/early disease course	MAS	ALT↑ AST↑ FER↑ FIB↓	Steroids Tocilizumab	Dexamethasone (15 mg/day)	Dexamethasone Canakinumab Ruxolitinib	2.5 mg bid→5 mg bid	6	Partial response	No
He et al. (2023) (15)	P4	Female	3	At sJIA onset/early disease course	Fever Arthritis	WBC↑ FER↑ ALT↑ AST↑ FIB↓ ESR↑ CRP↑	Steroids	Methylprednisolone pulse(25 mg/kg/day × 3 days) followed by prednisone(25 mg/day) MTX Tocilizumab	Prednisone Canakinumab MTX	2.5 mg bid→5 mg bid	4	Complete remission rebound effect	Upper respiratory tract infection
He et al. (2023) (15)	P5	Male	3.3	At sJIA onset/early disease course	Fever Systemic edema	PLT↓ ALT↑ AST↑ FER↑ FBI↓	Steroids Tocilizumab	Methylprednisolone pulse (25 mg/kg/day × 3 days) followed by Prednisone (40 mg/day) etoposide	Canakinumab MTX	2.5 mg bid→5 mg bid	3	Partial response, disease flare	Upper respiratory tract infection
Case1	P6	Male	1.6	At sJIA onset	Fever Rash Dorsal foot edema Coronary artery Respiratory failure Arthritis	WBC↑ ALT↑ AST↑ PLT↓ FIB↓ CRP↑ FER↑ TG↑	Methylprednisolone pulse (15 mg/kg/day→20 mg/kg/day) IVIG	Prednisone (20 mg/day)	Ruxolitinib	2.5 mg bid (0.5 mg/kg/day)	26 months at last follow-up	Complete remission	No
Case2	P7	Female	4	During treatment	Fever Rash Arthritis ARDS	WBC↑ PLT↓ ALT↑ AST↑ FER↑ TG↑ FIB↓	Methylprednisolone pulse (21.7 mg/kg/day) Tocilizumab	Plasma exchange Methylprednisolone pulse (21.7 mg/kg/day)	Ruxolitinib Tocilizumab	10 mg bid (0.87 mg/kg/day)	15 months at last follow-up	Complete remission	Neutropenia↓

MAS, macrophage activation syndrome; WBC, white blood cells; FER, ferritin; MTX, methotrexate; CRP, C-reactive protein; ESR, erythrocyte sedimentation rate; ALT, alanine transaminase; AST, aspartate aminotransferase; FIB, fibrinogen.

Normal pediatric reference ranges apply only to the two patients from our hospital: WBC 4.4–11.9 × 10⁹/L, PLT 188–472 × 10⁹/L, ESR < 20 mm/h, hs-CRP < 10 mg/L, SF 10–291 ng/mL, FIB 2.0–4.0 g/L, AST 14–44 U/L, ALT 7–30 U/L, LDH 120–250 U/L, TG 0–2.3 mmol/L.

Before initiation of ruxolitinib, all patients showed inadequate response to glucocorticoid therapy. Four patients had received high-dose methylprednisolone pulse therapy, five had been treated with tocilizumab, three with cyclosporine, and one had undergone plasma exchange, yet disease control remained unsatisfactory.At the time of ruxolitinib initiation, some patients continued to receive concomitant immunosuppressive or rescue therapies, including cyclosporine (3/7), tocilizumab (1/7), and plasma exchange (1/7).

Following ruxolitinib treatment, five of the seven patients achieved complete remission and two achieved partial remission. In the two patients with partial remission, clinical symptoms and inflammatory markers improved significantly but laboratory parameters did not fully normalize or disease activity fluctuated, thus not meeting criteria for complete remission. Patient 5 developed recurrent fever, tachypnea, and generalized edema on day 18 of ruxolitinib therapy. The drug was discontinued for two weeks to exclude a potential adverse reaction. The episode was subsequently attributed to MAS relapse, and ruxolitinib was restarted, leading to successful glucocorticoid withdrawal. Patient 3 experienced recurrence of fever and other MAS-related manifestations during glucocorticoid tapering. Both patients ultimately achieved complete remission after the addition of canakinumab. All patients received concomitant glucocorticoids at the time of ruxolitinib initiation, which were gradually tapered during follow-up. Glucocorticoids were discontinued within approximately 3 months in Patients 5 and 6, within 6 months in Patient 7, and were nearly discontinued within 4–6 months in Patients 3 and 4. The glucocorticoid tapering course was not clearly described for Patients 1 and 2 in the literature.

Regarding safety, one patient developed Epstein–Barr virus–associated hemophagocytic lymphohistiocytosis and agranulocytosis three weeks after starting ruxolitinib. The condition improved after drug discontinuation and plasma exchange. Among the remaining patients, one experienced reversible neutropenia and two developed upper respiratory tract infections. No other serious adverse events were reported.

## Discussion

MAS is the most severe and rapidly progressive complication of sJIA, driven by impaired cytotoxic function and IFN-*γ*–mediated uncontrolled inflammation. Multiple cytokines, including IFN-*γ*, IL-6, IL-2, IL-10, and GM-CSF, signal through the JAK-STAT pathway, forming an amplification loop (21). Compared with IL-1 or IL-6 inhibitors targeting a single cytokine, ruxolitinib inhibits JAK1/2, modulating downstream signaling of IFN-*γ*, IL-6, and multiple other JAK-dependent cytokines, thereby regulating inflammation at the signal integration level and offering a theoretical advantage in refractory sJIA-MAS (11, 22).

According to the 2024 EULAR/PReS recommendations for the management of Still's disease, which also apply to sJIA-MAS, high-dose glucocorticoids remain the cornerstone of therapy, and additional treatments such as anakinra, cyclosporine, or the IFN-*γ* inhibitor emapalumab should be considered, although emapalumab has not yet been approved in Europe. Moreover, the guideline highlights JAK inhibitors as emerging therapeutic options for patients with insufficient response to standard therapy (7). These recommendations provide a rationale for exploring ruxolitinib in refractory sJIA-MAS.

In our seven patients, all presented with persistent high fever and markedly elevated ferritin during MAS episodes. Some exhibited severe systemic involvement, including respiratory failure, coagulopathy, or coronary artery dilation, and inflammatory markers improved slowly or worsened during treatment. Ruxolitinib was mainly used as a second-line or salvage therapy and was generally well tolerated. One patient achieved rapid clinical and laboratory improvement within approximately one week using ruxolitinib alone, gradually reaching complete remission. Aside from this case, nearly all patients required combination therapy with canakinumab or tocilizumab to control sJIA activity. In one patient, early combination of ruxolitinib with plasma exchange during an ongoing MAS cytokine storm and respiratory failure resulted in rapid stabilization. In this case, plasma exchange was initiated for refractory MAS under limited availability of alternative targeted therapies. Previous studies suggest that when IL-1 or IL-6 inhibitors are insufficient, plasma exchange may help remove circulating inflammatory mediators and control hyperinflammatory MAS (13, 23). PE rapidly clears circulating mediators, while ruxolitinib suppresses downstream inflammatory amplification via JAK-STAT signaling; their combined effect may help quickly stabilize systemic inflammation and protect organ function. However, plasma exchange and intensive immunosuppression may both increase the risk of infection. During treatment, strict infection monitoring should be performed for the patient.

Recent studies indicate that ruxolitinib may be a promising option for refractory rheumatic disease–associated MAS in adults. Pediatric reports also suggest potential benefits in controlling sJIA-associated lung disease (sJIA-LD). Bader-Meunier et al. described a patient who developed sJIA-MAS complicated by LD; after ruxolitinib combined with prednisone, clinical and laboratory parameters normalized, steroids were tapered, and catch-up growth was observed (22).

In Case 2, pulmonary involvement occurred during the hyperinflammatory phase of MAS, presenting as acute bilateral interstitial pulmonary edema and small to moderate pleural effusion. The disease had an abrupt onset and was accompanied by markedly elevated inflammatory markers. Peripheral blood showed no eosinophilia or atypical lymphocytosis, and tests for herpesvirus reactivation (HHV-6 and EBV) were negative, suggesting that DRESS syndrome could be tentatively excluded (24). As the systemic inflammation was brought under control, the pulmonary lesions almost completely resolved within two weeks. Overall, these findings are more consistent with acute inflammation-related lung injury secondary to MAS rather than typical sJIA-associated lung disease (sJIA-LD). Importantly, most patients still required IL-1 or IL-6 inhibitors after acute-phase control to maintain sJIA activity, indicating that ruxolitinib is more suitable as bridging therapy during uncontrolled inflammation rather than as long-term monotherapy.

Patient 2 had received the IL-6 receptor antagonist tocilizumab prior to the onset of MAS, and CRP levels did not increase during the MAS episode. Tocilizumab blocks the IL-6 signaling pathway and markedly suppresses hepatic synthesis of acute-phase reactants such as CRP and ferritin, thereby masking the true intensity of systemic inflammation. In a multicenter retrospective study, Shimizu et al. (25) demonstrated that children with sJIA-associated MAS receiving tocilizumab showed a lower frequency of fever and significantly lower levels of CRP, ferritin, triglycerides, and fibrinogen compared with those not treated with the drug, resulting in reduced sensitivity of conventional MAS classification criteria in this population. Therefore, clinicians should recognize the potential for atypical manifestations of MAS under IL-6 blockade and remain vigilant to avoid missed or delayed diagnosis when laboratory parameters appear only mildly elevated.

Regarding safety, ruxolitinib is associated with an increased risk of CMV and EBV reactivation (26), and some patients experienced neutropenia or thrombocytopenia (20, 27). Given the immune dysregulation inherent to MAS, baseline evaluation including blood counts, liver function, and viral screening is recommended. Early monitoring and dose adjustment or temporary discontinuation should be considered in cases of severe infection or bone marrow suppression. Most reports describe ruxolitinib use for several weeks to months, followed by gradual tapering, reinforcing its role as bridging therapy rather than long-term monotherapy (16). After inflammation is controlled, IL-1 or IL-6–targeted therapy remains the mainstay.

This study has several limitations. First, the sample size was small, and many patients received concomitant immunosuppressive agents such as tocilizumab and methylprednisolone, with some also undergoing plasma exchange. Plasma exchange may alter the pharmacokinetics of ruxolitinib and other biologic agents by removing intravascular molecules, including albumin-bound drugs and immunoglobulins, which could in turn affect the evaluation of therapeutic efficacy. These factors make it challenging to accurately determine the true effect of ruxolitinib monotherapy. In addition, variations in the initial dose and treatment duration among reported cases have made the optimal dosing and tapering regimens uncertain. In a pediatric HLH trial, ruxolitinib was administered orally at 2.5, 5, or 10 mg every 12 h according to body weight (28). The study observed clinical improvement in most patients within a few days and reported relatively low toxicity compared with intensive chemotherapy. This experience may serve as a reference for dose selection in patients with sJIA-MAS. Finally, as the available evidence mainly derives from case reports, publication bias cannot be excluded. Therefore, multicenter prospective studies are warranted to further clarify the efficacy and safety of JAK inhibitors at different disease stages.

## Conclusion

Ruxolitinib may be an effective option for pediatric patients with sJIA complicated by MAS, particularly those with refractory or severe disease unresponsive to conventional therapies. Early combination strategies, including plasma exchange in selected severe cases, may facilitate rapid disease control.Careful monitoring for cytopenia and viral reactivation is essential during treatment. Larger prospective studies are needed to further evaluate the efficacy and long-term safety of ruxolitinib in this population.

## Data Availability

The data analyzed in this study is subject to the following licenses/restrictions: The datasets analyzed during the current study are not publicly available due to patient privacy restrictions, nor are they included in the manuscript or Supplementary Material. They are available from the corresponding author on reasonable request. Requests to access these datasets should be directed to Wei Zhang, gczhangwei@163.com.
