# Supplementary material for: Case Report: Ruxolitinib for systemic juvenile idiopathic arthritis complicated by macrophage activation syndrome: two pediatric cases and literature review
Source: Front Pediatr. 2026 May 28;14:1812770. doi: 10.3389/fped.2026.1812770 (PMC13254185; doi:10.3389/fped.2026.1812770)
Supplement: Supplementary file 1 [file Table1.docx]

| Parameter | Abbreviation | Reference range (pediatric) | Unit |
| --- | --- | --- | --- |
| White blood cell count | WBC | 4.4 – 11.9 | ×10⁹/L |
| Hemoglobin | HB | 112 – 149 | g/L |
| Platelet count | PLT | 188 – 472 | ×10⁹/L |
| Erythrocyte sedimentation rate | ESR | < 20 | mm/h |
| Neutrophils | NEUT | 81.9 | % |
| High‑sensitivity C‑reactive protein | hs‑CRP | < 10 | mg/L |
| Ferritin | SF | 10 – 291 | ng/ml |
| D‑dimer | D-D | < 0.55 | mg/L |
| Fibrinogen | FIB | 2.0 – 4.0 | g/L |
| Aspartate aminotransferase | AST | 14 – 44 | U/L |
| Alanine aminotransferase | ALT | 7 – 30 | U/L |
| Lactate dehydrogenase | LDH | 120 – 250 | U/L |
| Triglycerides | TG | 0 – 2.3 | mmol/L |
| Interleukin‑6 | IL‑6 | =< 7 | pg/mL |
| Interleukin‑10 | IL‑10 | 0-4.9 | pg/mL |
| Interferon‑γ | IFN‑γ | 0-17.3 | pg/mL |

Source / Note：Clinical Laboratory, Chengdu Women’s and Children’s Central Hospital
